# Learning style preferences of architecture and interior design students in Saudi Arabia: A survey

**DOI:** 10.1016/j.mex.2019.04.021

**Published:** 2019-04-24

**Authors:** Wafa Labib, Irene Pasina, Abdelhakim Abdelhadi, Goze Bayram, Mohammad Nurunnabi

**Affiliations:** a College of Engineering, Prince Sultan University, P.O. Box 66833, Riyadh 11586, Saudi Arabia; b College of Business Administration, Prince Sultan University, P.O. Box 66833, Riyadh 11586, Saudi Arabia; c St. Antony’s College, University of Oxford, Oxford, 62 Woodstock Road, Oxford OX2 6JF, UK

**Keywords:** Survey Questionnaire, Interior design, Architecture, Student preferences, Learning style, Questionnaire survey, Saudi Arabia

## Abstract

This method article examines the Felder and Soloman’s (1999) Index of Learning Styles (ILS) questionnaire in the context of Saudi Arabian higher education. Specifically, it aims at exploring the learning styles preferences among the students in Interior Design and Architecture program. Clustering approach is used by grouping students based on the similarities measure of their learning style. The findings reveal that students in both programs share learning style of Reflective, Intuitive, Verbal and Global at 57% or higher.

• Little research has focused on the Learning Styles preferences in higher education.
• There is no significant difference of learning style preferences between Interior Design and Architecture students.
• Reflecting learning style in university is reinforced by local elementary and high schools
• The data can be used by the scientific community to explore differences in learning style preferences (e.g. students of different genders).

## Graphical abstract

**Figure:**
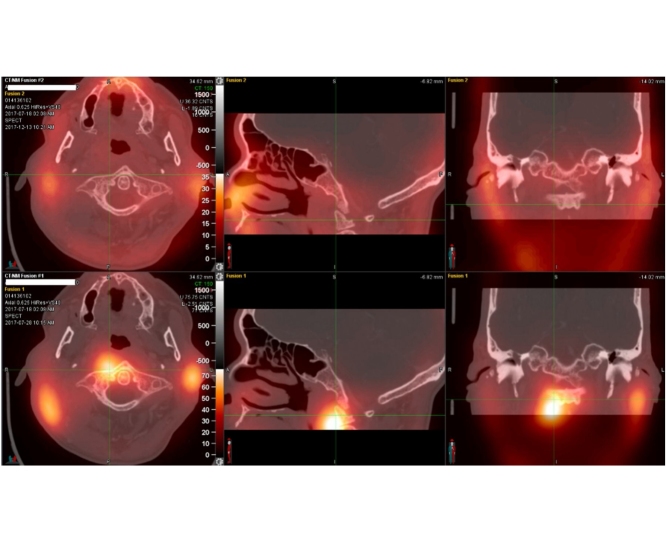

**Specification Table**

**Table:** 

Subject Area:	*Social Sciences*
More specific subject area:	*Higher Education*
Method name:	*Survey Questionnaire*
Name and reference of original method:	*Felder and Soloman’s (1999) Index of Learning Styles (ILS) questionnaire Available at: https://www.webtools.ncsu.edu/learningstyles/ (accessed 22 Jan 2019).*
Resource availability:	*The data is available in the article*

## Data and method details

In higher education, various teaching and learning styles have been observed (Felder and Soloman [1]). Keefe [2] defines learning style as “being characteristics of cognitive, affective and physiological behaviors that serve as relatively stable indicators of how learners perceive, interact with, and respond to learning environment”. According to Felder [3], learning style is defined as “characteristic, strengths and preferences in the way they take in and process information”. Felder and Brent [4] argue that students have different levels of motivation and different attitudes towards learning and teaching. Therefore, recognizing student learning preferences can enhance the ability of faculty to develop and design new learning opportunities (Massey et al. [5]) and, at the same time, helps students to improve their learning effectiveness in and outside the classroom (Dembo and Howard [6]). The traditional teaching methods include lectures, workshops, and tutorials for the presentation and discussion of ideas. However, the traditional teaching methods has turned out to be obsolete and were created outside the various discipline [8]. As indicated by Vassigh [8] “the utilization of such strategies does not fulfill the engineering students' needs and causes them numerous troubles in integrating concepts into their courses.” The integrating issues remained as a result of the pressure between imaginative reasoning (the creative side) and specialized perspectives (the engineering for example science and innovation) [9]. This was also supported by Ochshorn [10] who finds that there is a lack of integration of basic concepts in engineering students’ application. A study by Zhang and Sternberg [11] argue that many teachers had put accentuation on trying to enable students to learn more effectively to encourage effective learning for students. They propose that teachers could fluctuate their instructional techniques and to consider preferable student learning styles [11,12].

In the case of learning styles in engineering education, Nepal and Jenkins [7, p. 1] argue that teaching methods can be grouped “as traditional and modern, where the traditional teaching methods is defined as the conventional lectures followed by tutorial and/or laboratory sessions, while among modern methods, … the project-based learning (PBL) strategy that is acknowledged as a collaborative, progressive, student-centered, interactive, active and deep learning approach”.

This study examines Learning Styles preferences of architectural and interior design students in a private university in Saudi Arabia using the Index of Learning Styles (ILS) developed by Felder and Soloman [1].

## Data and sample

As mentioned earlier that the ILS survey was firstly introduced by Felder and Soloman [1] in 1999. The ILS works on the following four dimensions of learning styles:

• Sensing (concrete thinker, practical, oriented toward facts and procedures) or intuitive (abstract thinker, innovative, oriented toward theories and underlying meanings);
• Visual (prefer visual representation of resented material, such as pictures, diagrams, and flow charts) or verbal (prefer written and spoken explanations);
• Active (learn by trying things out, enjoy working in groups) or reflective (learn by thinking things through, prefer working alone or with a single familiar partner);
• Sequential (linear thinking process, learn in small incremental steps) or global (holistic thinking process, learn in large leaps).

There are 44 questions in the ILS questionnaire, Students were instructed to choose "a" or "b" for each question; if both "a" and "b" seem to apply, they should choose the one that applies more frequently to them. For instance, Q1 was: I understand something better after I: a) try it out. b) think it through.

The data were collected from the students of Architecture and the Interior Design program in a private university in Saudi Arabia. The target population was for female students since Architecture and the Interior Design program are offered for female students in that private university. All students were debriefed about anonymity and confidentiality. 150 questions were distributed and 92 usable questionnaires were collected representing 61.3% response rate (See Appendix A and B).

Both programs, there are four level of students: Freshman, Sophomore, Junior and Senior. At the Freshman level, there are some shared courses conceived to build a common core-knowledge in the design field. Starting from the Sophomore level, the courses are differentiated between the two programs. In this study, the main aim is to understand and evaluate the similarities and differences between Architecture students and Interior Design students throughout the four years.

In this study, the clustering approach was conducted on students as items and then on learning styles as variables. Hierarchal clustering techniques conducted either based on series of successive division of the items or based on successive merges. Agglomerative hierarchal methods start with individual objects, the most similar objects are first joined together and these groups, then merged together according to their similarity. While division hierarchal work in opposite way. The initial group is divided into two groups in the sense that the items in each group as far apart as it can be. The subgroups are then divided in the same approach. An agglomerative hierarchal method is used by applying complete linkage clustering. In complete linkage clustering, the similarities between clusters is found according to the similarity between the elements, one from each cluster that are most distant [13]. Minitab software package will be used to discover the natural grouping of students’ learning styles (or variables) is based on the results of Index of Learning Style questionnaire they provide [13]. The quantitative scale on which we measure their learning style is ranged from 1 to 11, as used by Felder and Soloman [1].

## Experimental design

Table 1 shows the representation of each learning style in numerical format as it appears in the dendrogram and cluster formation processes. Table 2 shows the result output of the variables (learning style) similarities among Architecture and Interior Design students using Minitab software package. It presents the formation of Learning styles variables based on the similarity level between variables (learning styles) as previewed from students’ point of view. For example, in architecture at step 1 Cluster 6 (Verbal) is joining Cluster 8 (Global) at 81.93% similarity level, while, cluster 6 is joining cluster 8 in the interior design at 81.32% level of similarity.

**Table 1:** Numerical representations of learning style.

Numerical Representation	Learning Style
1	Active
2	Reflective
3	Sensing
4	Intuitive
5	Visual
6	Verbal
7	Sequential
8	Global

**Table 2:** Learning style clusters for Architecture and Interior design students.

Architictrure Students						
Step	Number of	Similarity	Clusters		New	No. obs.
1	7	81.93	6	8	6	2
2	6	68.60	2	6	2	3
3	5	56.99	2	4	2	4
4	4	43.59	1	3	1	2
5	3	37.42	1	7	1	3
6	2	21.34	1	5	1	4
7	1	0.00	1	2	1	8

Interior Design Students						
Step	Number of	Similarity	Clusters		New	No. obs.
1	7	81.32	6	8	6	2
2	6	71.34	2	6	2	3
3	5	57.76	2	4	2	4
4	4	53.27	3	7	3	2
5	3	44.15	1	3	1	3
6	2	38.41	1	2	1	7
7	1	0.00	1	5	1	8

**Fig. 1:**
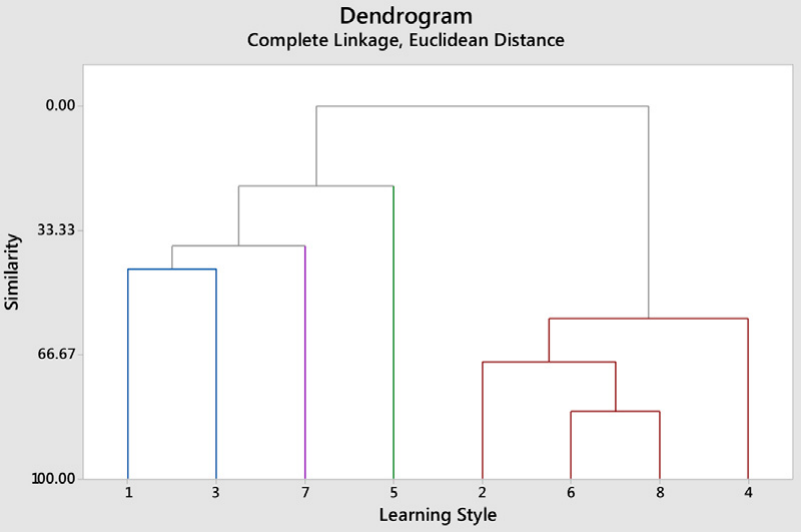
Complete linkage dendrogram for similarities among eight learning styles (Architecture students).

**Fig. 2:**
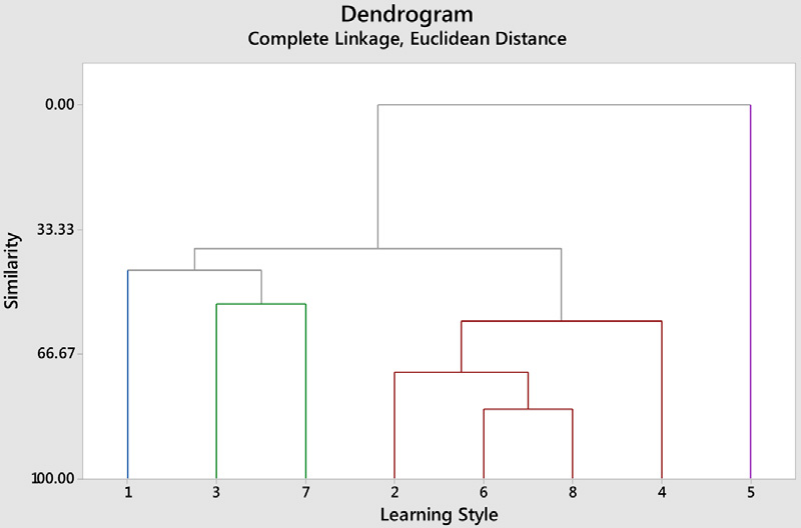
Complete linkage dendrogram for similarities among eight learning styles (Interior design students).

The dendrogram is formed to connect the learning style variables as shown in Fig. 1 for architecture students’ and Fig. 2 for Interior design students. Fig. 1 represents complete linkage dendrogram for similarities among eight learning styles of Architecture students whilst Fig. 2 shows complete linkage dendrogram for similarities among eight learning styles of Interior design students. This is because architecture students at 81.93% similarity level and interior design students at 81.32% similarity.

The results therefore indicated that there is no significant difference in learning styles between students of Architecture and Interior design. Students in both programs share learning style of Reflective, Intuitive, Verbal and Global at 57% or higher. While learning styles of Active, Sensing and sequential are shared at level of 43.59% or higher. When referring to questionnaire, it is clear that learning styles of Reflective, Intuitive, Verbal and Global are not students’ favorite and Active, Sensing and Sequential are the type of learners’ students are. Hence, instructors should deal with teaching methods aligned with this type of student [[14], [15], [16], [17], [18]].

## Conflict of interest

The authors declare no conflict of interest.

## Figures and Tables

**Table:** 

Student	Active	Reflective	Sensing	Intuitive	Visual	Verbal	Sequential	Global
1	0	3	0	3	9	0	1	0
2	0	5	3	0	5	0	1	0
3	0	5	0	1	5	0	5	0
4	5	0	9	0	9	0	0	0
5	3	0	5	0	9	0	7	0
6	7	0	7	0	5	0	11	0
7	9	0	0	3	7	0	5	0
8	3	0	5	0	7	0	3	0
9	1	0	5	0	3	0	3	0
10	1	0	0	3	3	0	1	0
11	9	0	0	1	7	0	1	0
12	5	0	0	5	3	0	0	1
13	1	0	3	0	3	0	1	0
14	7	0	7	0	11	0	5	0
15	3	0	9	0	7	0	0	3
16	1	0	7	0	3	0	3	0
17	3	0	3	0	5	0	5	0
18	0	3	0	3	0	1	1	0
19	1	0	3	0	7	0	3	0
20	0	0	3	0	5	0	9	0
21	0	5	5	0	7	0	0	5
22	0	1	0	7	5	0	3	0
23	3	0	9	0	9	0	5	0
24	7	0	3	0	5	0	1	0
25	0	1	0	5	5	0	5	0
26	0	1	0	3	5	0	1	0
27	3	0	7	0	9	0	0	1
28	0	5	0	3	9	0	0	3
29	0	1	0	1	5	0	3	0
30	1	0	3	0	3	0	1	0
31	0	1	0	0	5	0	5	0
32	1	0	0	7	9	0	0	1
33	0	5	5	0	9	0	0	1
34	0	1	1	0	3	0	7	0
35	0	1	0	1	3	0	5	0
36	1	0	0	5	5	0	1	0
37	1	0	0	1	5	0	3	0
38	3	0	7	0	1	0	7	0
39	1	0	1	0	3	0	0	0

**Table:** 

Student	Active	Reflective	Sensing	Intuitive	Visual	Verbal	Sequential	Global
1	11	0	0	5	11	0	0	5
2	1	0	1	0	9	0	7	0
3	3	0	7	0	1	0	1	0
4	3	0	0	7	9	0	0	1
5	0	5	11	0	5	0	1	0
6	9	0	0	3	11	0	1	0
7	7	0	0	1	11	0	0	3
8	0	3	5	0	11	0	7	0
9	0	1	1	0	3	0	7	0
10	1	0	0	1	7	0	3	0
11	7	0	7	0	9	0	9	0
12	7	0	9	0	9	0	0	5
13	0	1	0	1	7	0	3	0
14	0	3	0	1	9	0	1	0
15	5	0	3	0	9	0	1	0
16	7	0	5	0	9	0	7	0
17	0	3	0	9	7	0	3	0
18	5	0	0	5	11	0	3	0
19	7	0	0	7	11	0	5	0
20	1	0	3	0	11	0	0	1
21	0	5	3	0	7	0	5	0
22	1	0	3	0	7	0	1	0
23	0	1	0	1	7	0	1	0
24	3	0	3	0	5	0	0	3
25	3	0	3	0	9	0	7	0
26	0	3	1	0	1	0	5	0
27	3	0	0	7	11	0	0	3
28	0	1	1	0	3	0	0	3
29	7	0	0	3	3	0	7	0
30	0	7	5	0	7	0	3	0
31	9	0	3	0	9	0	3	0
32	3	0	1	0	3	0	3	0
33	1	0	7	0	5	0	1	0
34	5	0	0	5	9	0	0	1
35	0	7	3	0	7	0	0	3
36	3	0	5	0	0	**1**	3	0
37	3	0	5	0	9	0	1	0
38	1	0	1	0	3	0	1	0
39	5	0	0	3	9	0	0	1
40	1	0	0	5	7	0	0	1
41	9	0	1	0	7	0	9	0
42	0	1	0	3	7	0	1	0
43	0	3	0	5	7	0	1	0
44	0	3	0	7	9	0	0	1
45	0	1	9	0	7	0	3	0
46	1	0	5	0	9	0	5	0
47	3	0	0	7	11	0	0	3
48	3	0	0	3	11	0	5	0
49	7	0	1	0	3	0	3	0
50	0	1	5	0	9	0	0	1
51	0	3	5	0	9	0	1	0
52	0	3	0	7	0	**1**	0	3
53	5	0	1	0	7	0	3	0

## References

[1] Felder R.M., Soloman B.A. (1999). Index of Learning Styles Questionnaire. https://www.webtools.ncsu.edu/learningstyles/.

[2] Keefe J.W., Keefe J.W. (1979). Learning style: an overview. Students Learning Styles: Diagnosing and Prescribing Programs.

[3] Felder R.M. (1996). Matters of style. ASEE Prism..

[4] Felder R.M., Brent R. (2005). Understanding student differences. J. Eng. Educ..

[5] Massey M.G., Kim S.H., Mitchell C. (2011). A study of the learning styles of undergraduate social work students. J. Evid.-Based Soc. Work.

[6] Dembo M.H., Howard K. (2007). Advice about the use of learning styles: a major myth in education. J. Col. Read. Learn..

[7] Nepal K.P., Jenkins G.A. (2019). Blending project-based learning and traditional lecture-tutorial based teaching approaches in engineering design courses. Proceedings of the 2011 AAEE Conference.

[8] Vassigh S. (2005). A Comprehensive Approach to Teaching Structures Using Multimedia. AIA Report on University Research.

[9] Fahmi M., Abdul Aziz A. (2012). The integration of structural knowledge in studio design projects, an assessment curriculum in: architecture course in SUST. J. Sci. Technol..

[10] Ochshorn J. (1991). Teaching Technology: What Do Architects Need to Know About Structures? ACS Technology Conferences: The City and Technology Los Angeles.

[11] Zhang L., Sternberg R. (2005). A threefold model of intellectual styles. Educ. Psychol. Rev..

[12] Chen N., Kinshuk W., Chun W., Liu C. (2011). Effects of matching teaching strategy to thinking style on learner’s quality of reflection in an online learning environment. Comp. Educ..

[13] Abdelhadi A., Nurunnabi M. (2019). Engineering student evaluation of teaching quality in Saudi Arabia. Int. J. Eng. Educ..

[14] Sternberg R. (1994). Allowing for thinking styles. Educ. Lead..

[15] Hadden C. (2005). Making Connections: Instructional Strategies. http://education.uregina.ca/iteachered/modules/preservice/module3.html.

[16] Simelane S., Mji A. (2014). Impact of technology-engagement teaching strategy with the aid of clickers on student’s learning. Procedia Soc. Behav. Sci..

[17] Boese T. (2011). Comparison of selected teaching strategies incorporating simulation and student outcomes. Clin. Simul. Nurs..

[18] Tulbure C. (2012). Learning styles, teaching strategies and academic achievement in higher education: a cross-sectional investigation. Procedia Soc. Behav. Sci..

